# Review of molecular biological studies on acute lymphoblastic leukemia treated by modified shengmaiyin

**DOI:** 10.1097/MD.0000000000034013

**Published:** 2023-06-09

**Authors:** Dongyu Guo, Mengyu Gu, Fuquan Du, Yuting Zhao, Mingjie Gao, Jing Hao

**Affiliations:** a Heilongjiang University of Traditional Chinese Medicine, Harbin, China; b The First Affiliated Hospital of Heilongjiang University of Traditional Chinese Medicine, Harbin, China.

**Keywords:** acute lymphoblastic leukemia, modified shengmaiyin, molecular mechanism, network pharmacology

## Abstract

The objective was to explore the pharmacological mechanism of modified shengmaiyin (MSMY) in the treatment of acute lymphoblastic leukemia (ALL) by network pharmacology analysis. The effective components and predicted targets of MSMY were collected from TCMSP and Swiss target prediction databases, and the related targets of ALL were screened by GeneCards and DisGeNET. The core targets and related signaling pathways of MSMY active ingredients for the treatment of ALL were predicted by protein-protein interaction network (PPI), gene ontology (GO) and Kyoto Encyclopedia of Genes and Genomes (KEGG) functional enrichment analysis. We identified 172 potential targets for the active components of MSMY, 538 disease targets associated with ALL, and 59 common gene targets. PPI network showed that 27 targets such as triptolide, RAC-alpha serine/threonine-protein kinase (AKT1), vascular endothelial growth factor A and Caspase-3 (CASP3) were the core targets. KEGG enrichment analysis related signaling pathways included cancer pathway, phosphatidylinositol 3 kinase, PI-3K/protein kinase B (PI3K-Akt) signaling pathway, apoptosis and mitogen-activated protein kinase (MAPK) signaling pathway and IL-17 signaling pathway. The effective active components and potential therapeutic targets of MSMY in the treatment of ALL were initially identified by comprehensive network pharmacology, which provides a theoretical basis for further study of the material basis and molecular mechanism of MSMY in the treatment of ALL.

## 1. Introduction

Acute lymphoblastic leukemia (ALL) refers to the abnormal differentiation of lymphocytes in the early stage, resulting in malignant proliferation and abnormal blood function. The pathological manifestation of ALL is that a large number of blast cells gather in the bone marrow and destroy the normal hematopoietic function of the bone marrow, resulting in the symptoms of anemia, infection and hemorrhage, and extramedullary infiltration can occur in the late stage. ALL accounts for about 80% of childhood leukemia, with the highest incidence in children aged 1 to 4 years.^[1]^ At present, chemotherapy is the first recommended treatment for ALL. With the progress of modern chemotherapy technology, the outcome of ALL patients has been significantly improved, and the 5-year overall survival rate has been significantly improved. The 5-year overall survival rate of children with ALL can reach 90%.^[2]^ However, the adverse reactions caused by chemotherapy drugs, such as gastrointestinal reactions caused by chemotherapy and affecting the metabolism of nutrients in the body, can cause malnutrition. The bone marrow suppression caused by ALL chemotherapy can lead to the decline of immune function, secondary infection, bleeding, etc, so that children cannot tolerate chemotherapy, reduce the quality of life, and even have to interrupt chemotherapy, leading to treatment failure, and the related side effects seriously limit the clinical use of chemotherapy.^[3]^ Traditional Chinese Medicine (TCM) classifies ALL as consumptive disease, blood syndrome, malignant nucleus and so on according to different clinical manifestations, and holds that its pathogenesis is warm toxin, pathogenic toxin attacking and touching, pathogenic toxin burning nutrient yin, torturing viscera essence and blood, and damaging bone marrow. At the same time, studies have found that TCM treatment of ALL plays a greater role in inhibiting proliferation, promoting apoptosis, inducing differentiation, reversing drug resistance, enhancing efficacy and attenuating toxicity, and regulating immune function. Moreover, TCM has been widely recognized in prolonging the survival time of patients with ALL, reducing adverse reactions, enhancing therapeutic effect and reversing drug resistance.^[4]^

Modified shengmaiyin (MSMY) is composed of Shengmai Powder, Radix Glycyrrhizae, Radix Astragali, Herba Hedyotidis Diffusae, Herba Scutellariae Barbatae, Herba Solani Nigri and Radix Ampelopsis. Shengmai Powder was first recorded in Yi Xue Qi yuan and recorded in Leukemia Treatment of TCM. It has the effect of replenishing qi and nourishing yin. Modern pharmacological studies have shown that Shengmai Powder can improve the immune function of ALL patients and promote bone marrow hematopoiesis. It also protects the heart, liver, brain and other important organs, and reduces the myocardial toxicity caused by chemotherapy drugs.^[5]^ TCM network pharmacology, as a new mode of modern TCM research and development, contains the holistic concept of TCM, including TCM unique diagnosis and treatment ideas and rich clinical experience, and constitutes a research mode characterized by “network and “system.”^[6]^ A large number of clinical studies have shown that MSMY has a significant effect on ALL. However, the mechanism of action of MSMY in the treatment of ALL is still unclear. Therefore, in this study, network pharmacology was used to explore the possible mechanism of MSMY in the treatment of ALL, and to provide new theoretical support for the clinical treatment of ALL.

## 2. Materials and methods

### 2.1. MSMY active ingredient screening and target prediction

The active components of MSMYs were retrieved by using TCM Systematic Pharmacology Database and Analysis Platform (TCMSP, https://old.tcmsp-e.com/tcmsp.php) as the screening criteria, combined with literature data mining and data integration, and the targets of related active components were predicted. Oral Bioavailability (OB) and Drug Likeness index were used as the relevant indicators for screening, and the chemical components with OB ≥ 30% and Drug Likeness ≥ 0.18 in the database were set as candidate active ingredients and their target proteins were obtained. TCMSP and Swiss target prediction database (http://www.swisstargetprediction.ch/) were used for active ingredient target prediction. At the same time, the UniProt database (https://www.uniprot.org/) established for human species was used to query the screening targets, and the genes of these targets were normalized.

### 2.2. Screening of disease-related targets

ALL-related targets were obtained by searching GeneCards (https://www.genecards.org/) and DisGeNET (https://www.disgenet.org/), using the keyword “acute lymphoblastic leukemia,” GeneCards were screened for Relevance score ≥ 20. To remove the duplicate gene targets, UniProt database was used to query the screening targets, and the above targets were normalized.

### 2.3. Screening of common diseases and drug targets

The Venny 2.1 online mapping platform (https://bioinfogp.cnb.csic.es/tools/venny/index.html) was used to screen the targets of “acute lymphoblastic leukemia” and “MSMY,” and the intersection targets of ALL and MSMY were obtained. At the same time, the intersection target points are visualized and analyzed.

### 2.4. Protein-protein interaction (PPI) network construction of intersection target

PPI network can reveal the topological structure characteristics and functional organization mechanism of protein complexes and functional modules in biological processes (BP) and clarify the molecular correlation between diseases and drugs. Import the obtained intersection targets into the STRING11.5 database (https://cn.string-db.org/), the species was defined as Homo sapiens, the protein interaction was obtained, the interaction threshold was set as “highest confidence” (>0.9), and the free protein in the results was hidden to obtain the PPI relationship between MSMY and ALL. Then the PPI network diagram was drawn by Cytoscape v3.9.1 software to screen the core targets, and the data of the core targets were visualized to construct the PPI network diagram.

### 2.5. Gene ontology (GO) and Kyoto encyclopedia of genes and genomes (KEGG) enrichment analysis

For the core targets of the screening, the DAVID data platform (https://david.ncifcrf.gov/) was used for GO functional annotation and KEGG pathway enrichment analysis. Select “Homo species” on the DAVID platform; To further analyze the BP, cellular component, molecular function and signaling pathway of MSMY related to ALL treatment. The results were visualized and analyzed by bioinformatics online platform (http://bioinfo.seu.edu.cn/xxpt/).

## 3. Result

### 3.1. Analysis of active ingredients and action targets of MSMY

Through TCMSP database search, 212 active ingredients were screened out, including 22 ginseng, 9 Schisandra chinensis, 20 Astragalus membranaceus, 7 Hedyotis diffusa, 29 Scutellaria barbata, 7 Solanum nigrum, 11 Ampelopsis japonica and 93 Glycyrrhiza uralensis; Because the chemical constituents of Ophiopogon japonicus could not be retrieved from TCMSP database, the chemical constituents of Ophiopogon japonicus were retrieved from chemical professional database and screened from Swiss ADME database, and 7 effective components were obtained. The results showed that these different drugs contained common active ingredients, as shown in Table 1.

**Table 1:** MSMY common active ingredient information table.

Number	Mol ID	Molecule name	OB%	DL	Drug
1	MOL000098	quercetin	46.43	0.28	Radix Ampelopsis, Herba Scutellariae Barbatae, Radix Glycyrrhizae, Radix Astragali, Herba Solani Nigri, Radix Ophiopogonis, and Herba Hedyotidis Diffusae.
2	MOL000449	Stigmasterol	43.83	0.76	Radix Ampelopsis, Herba Scutellariae Barbatae, Radix Ophiopogonis, Radix Ginseng, Herba Hedyotidis Diffusae
3	MOL000358	beta-sitosterol	36.91	0.75	Radix Ampelopsis, Herba Scutellariae Barbatae, Radix Ginseng, Herba Hedyotidis Diffusae
4	MOL000359	sitosterol	36.91	0.75	Radix Ampelopsis, Herba Scutellariae Barbatae, Radix Glycyrrhizae, Herba Solani Nigri
5	MOL000953	CLR	37.87	0.68	Herba Scutellariae Barbatae, Herba Solani Nigri
6	MOL000211	Mairin	55.38	0.78	Radix Glycyrrhizae, Radix Astragali
7	MOL000354	isorhamnetin	49.6	0.31	Radix Glycyrrhizae, Radix Astragali
8	MOL000239	Jaranol	50.83	0.29	Radix Glycyrrhizae, Radix Astragali
9	MOL000417	Calycosin	47.75	0.24	Radix Glycyrrhizae, Radix Astragali
10	MOL000392	formononetin	69.67	0.21	Radix Glycyrrhizae, Radix Astragali
11	MOL000422	kaempferol	41.88	0.24	Radix Glycyrrhizae, Radix Astragali, Radix Ginseng
12	MOL005317	Deoxyharringtonine	39.27	0.81	Radix Ginseng, Fructus Schisandrae

DL = drug likeness, MSMY = modified shengmaiyin, OB = oral bioavailability.

Cytoscape v3.9.1 software was used to screen MSMY for active ingredients with degree ≥ 10. As shown in Table 2, 12 main components were obtained by using the software, including quercetin, Ophiopogon dihydroisoflavone E, homophyllindines, kaempferol, baicalein, Aristolochia mupinensis amide, N-coumaroyl tyramine, licochalcone A, red door blue phenol, Ophiopogon dihydroisoflavone B, β-carotene and naringenin.

**Table 2:** Chemical information table of the main active ingredients of MSMY.

Number	Mol ID	Molecule name	Degree	Drug
1	MOL000098	quercetin	62	Radix Ampelopsis, Herba Scutellariae Barbatae, Radix Glycyrrhizae, Radix Astragali, Herba Solani Nigri, Radix Ophiopogonis, and Herba Hedyotidis Diffusae.
2	MOL000006	Ophiopogonanone E	43	Ophiopogon japonicus
3	MOL000173	Dinatin	18	Scutellaria barbata
4	MOL000422	kaempferol	15	Radix Glycyrrhizae, Radix Astragali, Radix Ginseng
5	MOL002714	baicalein	14	Scutellaria barbata
6	MOL000002	Moupinamide	14	Ophiopogon japonicus
7	MOL000001	n-coumaroyltyramine	12	Ophiopogon japonicus
8	MOL000497	licochalcone a	12	Licorice
9	MOL000004	Orchinol	11	Ophiopogon japonicus
10	MOL000003	Ophiopogonone B	10	Ophiopogon japonicus
11	MOL002773	beta-carotene	10	Nightshade
12	MOL004328	naringenin	10	Licorice

MSMY = modified shengmaiyin.

**Figure 1.**
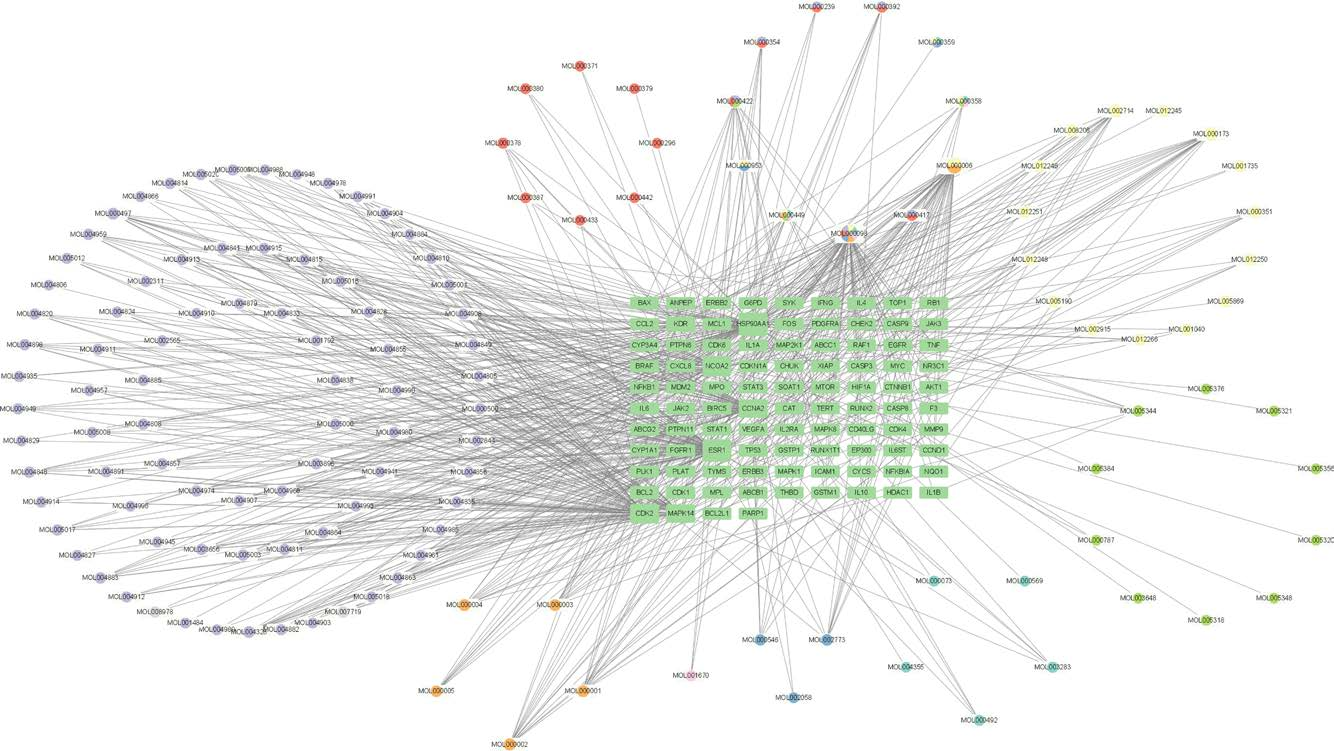
Active component-target network of MSMY. The circle is the active ingredient of the drug; the green square is the target spot. The purple round is Glycyrrhiza uralensis; the orange round is Ophiopogon japonicus; the red round is Astragalus membranaceus; the yellow round is Scutellaria barbata; the green round is Panax ginseng; the cyan round is Ampelopsis japonica; the blue round is Solanum nigrum; the pink round is Hedyotis diffusa; and the multicolor round is an intersection component. MSMY = modified shengmaiyin.

A total of 230 different drug targets were screened in TCMSP database and Swiss target prediction database, including 13 ginseng, 9 Schisandra chinensis, 57 Ophiopogon japonicus, 14 Astragalus membranaceus, 10 Hedyotis diffusa, 21 Scutellaria barbata, 10 Solanum nigrum, 9 Ampelopsis japonica and 87 Glycyrrhiza uralensis. And standardized by the UniProt database. The WSMY potential active ingredients and potential targets for the treatment of ALL were imported into Cytoscape v3.9.1 software to construct the component-target network diagram of TCM,as shown in Figure 1.

### 3.2. Relevant targets of ALL

**Figure 2.**
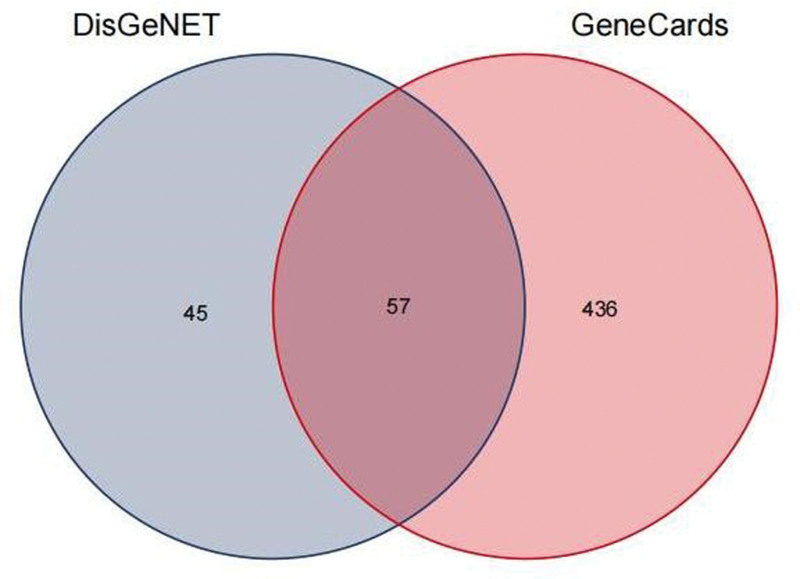
Venn diagram for screening of potential targets in ALL. ALL = acute lymphoblastic leukemia.

A total of 538 ALL-related targets were obtained by applying Gene Cards and DisGeNET databases to screen ALL-related targets and deleting duplicate targets, as shown in Figure 2.

### 3.3. Intersection target of compound active ingredient target and disease target

**Figure 3.**
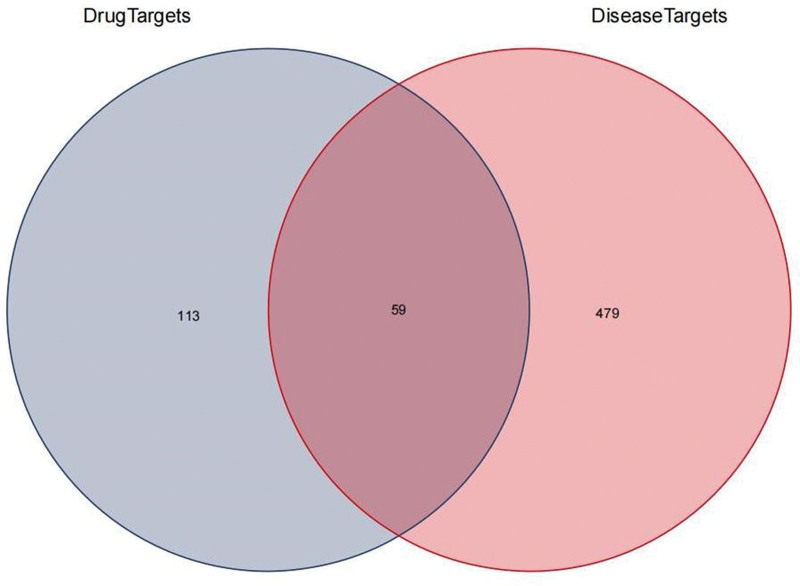
Venn plot of compound active ingredient targets and disease targets.

The 172 targets screened by MSMY and 538 targets of ALL were compared and analyzed to obtain 59 intersection targets of MSMY and ALL, and the Wayne diagram was drawn, as shown in Figure 3.

### 3.4. PPI network construction

**Figure 4.**
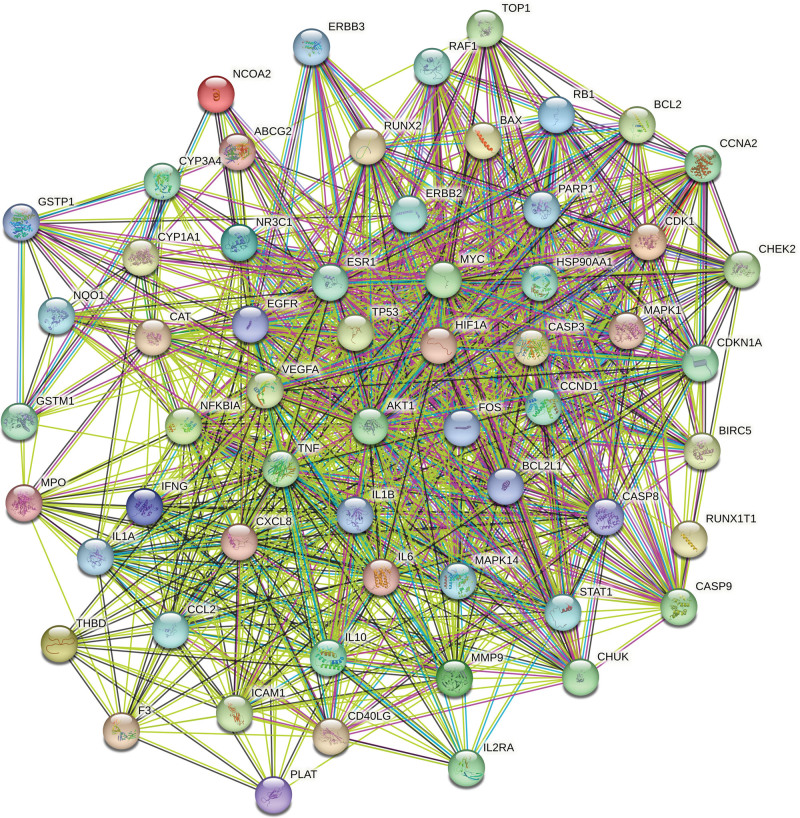
Intersection target PPI network view. PPI = protein-protein interaction.

**Figure 5.**
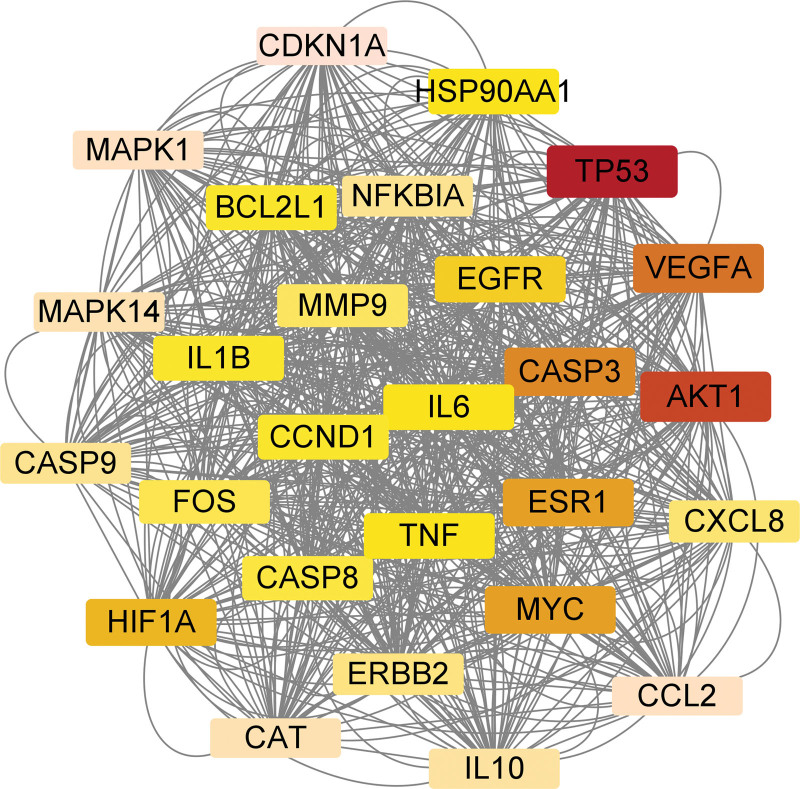
Core target PPI network. As shown in the figure, the redder the color, the more important it is in the network. PPI = protein-protein interaction.

The 59 selected intersection targets were imported into the STRING11.0 platform to construct the PPI network diagram. Homo sapiens was defined as the species, and the protein interaction was obtained. During the acquisition process, the minimum interaction threshold of the protein target was set as “highest confidence” (>0.4), and other settings were default settings. The PPI network relationship was obtained by hiding the free protein in the results, as shown in Figure 4. The network data is imported into Cytoscape v3.9.1 software for topology analysis, and 59 nodes and 877 edges are obtained. The median of “degree ≥ 66,” was used to screen the core targets. Therefore, 27 core targets of MSMY for ALL were obtained, as shown in Table 3. At the same time, Cytoscape v3.9.1 software was used for visual analysis of the core targets, as shown in Figure 5.

**Table 3:** Core target information table.

Target	Degree	Betweenness centrality (BC)	Closeness centrality(CC)
TP53	108	206.1592002	0.935483871
AKT1	102	109.5395024	0.892307692
VEGFA	98	119.9762889	0.865671642
CASP3	96	65.23871914	0.852941176
MYC	94	74.7131493	0.84057971
ESR1	94	95.58613884	0.84057971
HIF1A	92	63.61756928	0.828571429
EGFR	90	75.20509014	0.816901408
TNF	88	73.30972562	0.805555556
IL6	88	64.8369484	0.805555556
HSP90AA1	88	96.22802522	0.805555556
BCL2L1	86	39.32181492	0.794520548
IL1B	86	54.60943884	0.794520548
CCND1	86	40.28292015	0.794520548
CASP8	84	35.12878482	0.783783784
FOS	82	40.20333581	0.773333333
MMP9	80	43.1456125	0.763157895
CXCL8	78	39.0135849	0.753246753
ERBB2	76	31.47401809	0.743589744
CASP9	74	20.27110313	0.734177215
NFKBIA	74	18.62779106	0.734177215
IL10	72	20.81062322	0.725
CAT	70	31.67836783	0.716049383
MAPK14	70	17.15471792	0.716049383
MAPK1	68	21.61436389	0.707317073
CCL2	68	22.60649702	0.707317073
CDKN1A	66	13.67684771	0.698795181

AKT1 = RAC-alpha serine/threonine-protein kinase, CASP3 = Caspase-3, ESR1 = estrogen receptor 1, MAPK = mitogen-activated protein kinase, MYC = Myc proto-oncogene protein, TP53 = triptolide, VEGFA = vascular endothelial growth factor A.

### 3.5. GO and KEGG enrichment analysis of intersection targets

**Figure 6.**
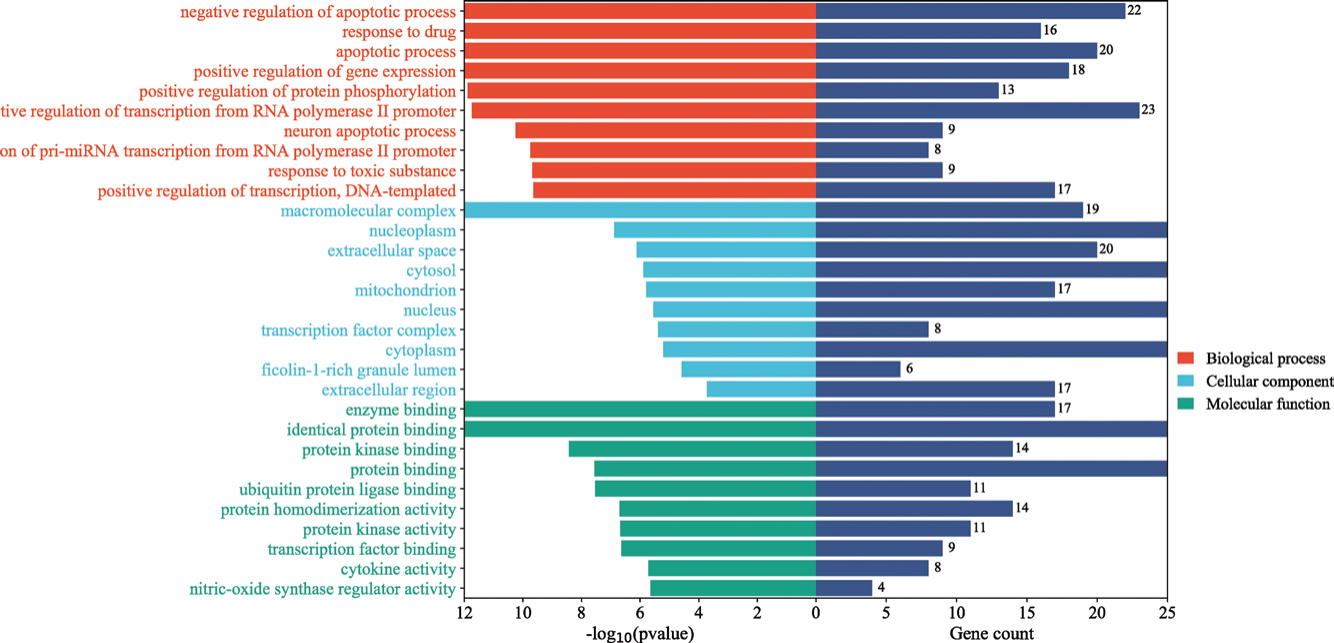
GO functional enrichment analysis. GO = gene ontology.

The GO functional enrichment analysis of 59 intersected core targets was performed on the DAVID online platform, and 567 GO enrichment items were obtained, including 454BP, 36CC, and 77MF. According to the number of enriched genes, the top 10 items were selected for visual analysis, as shown in Figure 6. The results showed that MSMY treatment of ALL mainly involved BP, such as drug response, apoptosis process, negative regulation of apoptosis process, positive regulation of gene expression, positive regulation of protein phosphorylation and response to toxic substances. These targets bind to enzymes, proteins, transcription factors, cytokines and proteins through molecular functions, and have related effects on nuclear plasma, cytoplasm, mitochondria, transcription factor complexes and cytoplasm, as well as extracellular matrix components.

**Figure 7.**
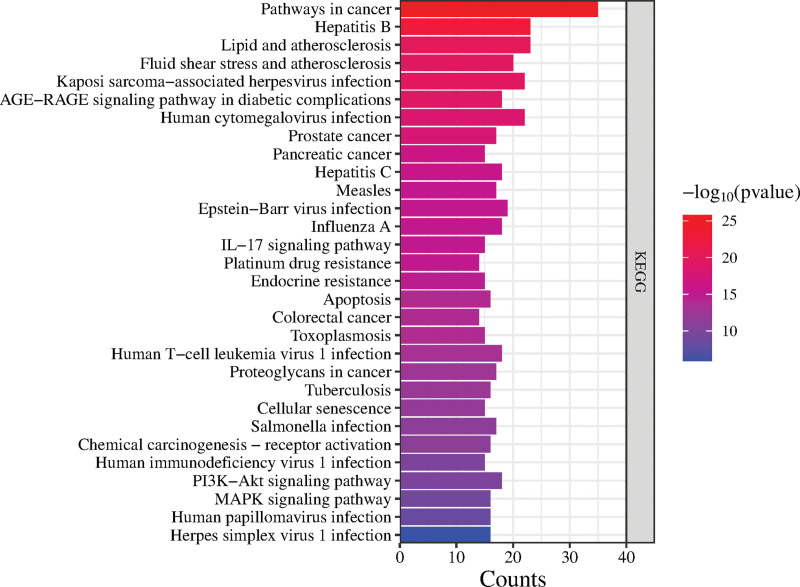
KEGG pathway analysis of core targets. KEGG = Kyoto encyclopedia of genes and genomes.

The DAVID online platform was used to analyze the KEGG pathway enrichment of core intersection targets, and genes were enriched in 143 signaling pathways. According to the gene enrichment ranking, 30 signaling pathways were selected for visual analysis, as shown in Figure 7. In addition, the results in Figure 7 indicate that the treatment of ALL by MSMY may be mainly related to the cancer pathway, phosphatidylinositol 3 kinase, PI-3K/protein kinase B (PI3K-Akt) signaling pathway, apoptosis and mitogen-activated protein kinase (MAPK) signaling pathway, and IL-17 signaling pathway.

## 4. Discussion

### 4.1. Research progress of ALL in Chinese and Western medicine

ALL is the most common type of leukemia with abnormal proliferation of hematopoietic stem cells. It is also a tumor disease with high incidence in children and adolescents. At present, it is divided into 3 types according to the severity of the disease: low-risk, intermediate-risk and high-risk. The pathogenesis of ALL is complex, involving multiple factors.^[7]^ ALL generally responds well to chemotherapy, and current dose-intensification strategies for ALL can significantly improve patient mortality, with 5-year overall survival currently estimated at 85% in developed countries and 35% in developing countries. However, early mortality, high side effects of chemotherapy and high recurrence rate are still the main problems.^[8]^

TCM has outstanding performance in the synergistic treatment of ALL, which can improve the side effects of chemotherapy and improve the quality of life of patients. Moreover, ALL is classified into the category of “consumptive disease” and “blood syndrome” according to the clinical manifestations of TCM, with the accumulation of pathogenic toxin as the main feature, which can be divided into 4 types: deficiency of both qi and yin, excessive heat toxin, phlegm nucleus and accumulation of symptoms. Among them, about 90% of ALL is the accumulation of pathogenic toxin and deficiency of both qi and yin.^[9]^ Professor Sun Weizheng, Department of Hematology, the First Affiliated Hospital of Heilongjiang University of Traditional Chinese Medicine, believes that ALL is due to congenital deficiency or acquired malnutrition, the 6 exogenous pathogens cause the deficiency of viscera, and the toxin enters by taking advantage of the deficiency, causing qi stagnation and blood stasis in the human body, phlegm and blood stasis form the disease, and internal deficiency is the root cause, so the treatment should be based on the rise and fall of pathogens and healthy qi to “strengthen the healthy qi” and “eliminate pathogens.” Through the above understanding, Sun Weizhengjiao created MSMY on the basis of Shengmai San through many years of clinical experience and clinical efficacy observation. The prescription is composed of ginseng, Schisandra chinensis, Ophiopogon japonicus, Astragalus membranaceus, licorice, Hedyotis diffusa, Scutellaria barbata, Solanum nigrum and Ampelopsis japonica. Its efficacy is to benefit qi and promote body fluid, clear heat and detoxify. Radix Astragali, Radix Ginseng, Fructus Schisandrae Chinensis, Radix Ophiopogonis, etc. Are compatible to regulate and invigorate qi, blood, yin and Yang; Herba Hedyotidis Diffusae, Herba Scutellariae Barbatae, Herba Solani Nigri, Radix Ampelopsis, etc. Clear away heat and toxic materials; Radix Glycyrrhizae tonifies deficiency, regulates the middle warmer, and harmonizes all medicines in the whole prescription. Modern pharmacological studies have found that Shengmaisan can improve the immune function of ALL patients and promote bone marrow hematopoiesis. At the same time, studies have found that Astragalus polysaccharide plays an important role in promoting hematopoiesis, enhancing immunity and regulating inflammation. Herba Hedyotidis Diffusae and Herba Scutellariae Barbatae are widely used in various tumors. The main anti-tumor active components (flavonoids and anthraquinones) of Herba Hedyotidis Diffusae can induce apoptosis of leukemia cells, increase the specific immune function and nonspecific immune function of the body, and have a certain attenuation effect on tumor chemotherapy drugs. Diterpenoids, flavonoids and polysaccharides in Scutellaria barbata have the effect of treating leukemia by inhibiting the proliferation of tumor cells, inhibiting the formation and metastasis of tumor cells, regulating immunity, and inducing the apoptosis of tumor cells.^[10]^

### 4.2. Study on the mechanism of MSMY in the treatment of ALL

Twelve main active components of MSMY in the treatment of ALL were screened out by TCM compound network pharmacology research method, comprising quercetin, Ophiopogon dihydroisoflavone E, homophylline, kaempferol, baicalein, muping aristolochic acid amide, N-coumaroyl tyramine, licochalcone A, red door blue phenol, Ophiopogon dihydroisoflavone B, beta-carotene and naringenin.

Among them, quercetin, Ophiopogon japonicus dihydroisoflavone E, homoplantain, kaempferol and baicalein are all flavonoids. Flavonoids have the effects of inhibiting proliferation, inducing apoptosis, blocking cycle, inhibiting angiogenesis, increasing the production of reactive oxygen species and inhibiting tubulin function in leukemia cells. Modern pharmacological studies have shown that quercetin can inhibit platelet aggregation, significantly inhibit platelet aggregation induced by thrombin and platelet activating factor, significantly inhibit the effect of cancer promoters, inhibit the growth of malignant cells in vitro, resist tumors, inhibit the expression of inflammatory mediators, and mediate cell signal transduction; Quercetin can inhibit the malignant proliferation of leukocytes, restore the hemogram to a relatively normal level, and has a significant inhibitory effect on ALL.^[11,12]^ A number of studies have found that dihydroisoflavone E of Ophiopogon japonicus shows a variety of pharmacological activities, such as cardiovascular protection, anti-inflammatory, anti-cancer, antioxidant, immunomodulation, etc.^[13]^; Homoplanin can play an anti-tumor role by inhibiting tumor cell proliferation, inducing tumor cell apoptosis, invasion and metastasis, tumor angiogenesis and other mechanisms, has multiple targets and a wide anti-tumor spectrum, plays a role in a variety of tumors, has weak toxicity to normal cells. At the same time, the combination of homo-plantain and a variety of anti-tumor drugs can significantly enhance the cytotoxicity of anti-tumor drugs to tumor cells.^[14]^ Kaempferol is the most effective scavenger of active oxygen free radicals,^[15]^ but its cytostatic effect and apoptosis-mediated effect are not as good as those of kaempferol.

In this study, 27 core targets were predicted for MSMY in the treatment of ALL, including triptolide, RAC-alpha serine/threonine-protein kinase (AKT1), vascular endothelial growth factor A, Caspase-3 (CASP3), Myc proto-oncogene protein and estrogen receptor 1. Among them, triptolide is a powerful tumor suppressor and one of the popular targets for the treatment of cancer. Activation of p53 can play a certain role in anti-cancer. P53 gene can play a role in inducing apoptosis of leukemia cells by inhibiting the activity of telomerase reverse transcriptase and telomerase and inhibiting the expression of Bcl-2 gene.^[16]^ Glucocorticoid resistance is a major driver of treatment failure in ALL, and AKT1 kinase acts as a major negative regulator of glucocorticoid receptor protein activity.AKT1 impairs glucocorticoid-induced gene expression by directly phosphorylating the glucocorticoid receptor at the S134 position and blocking glucocorticosteroid-induced translocation of the receptor to the nucleus.^[17]^Vascular endothelial growth factor A regulates the transendothelial migration of microvascular endothelial cells, VEGF mediates leukemic cell entry into the central nervous system and leptomeningeal infiltration, and VEGF plays a role by activating the VGEFR family to regulate vascular growth and protect leukemic cells from apoptosis.^[18]^Apoptosis is a kind of normal death under the control of relevant genes in order to maintain the homeostasis of cells. The proteins involved in the process of apoptosis include caspases and Bcl-2 (IAPs). The mode of action of CASP3 is that when cells are damaged, it mainly shows mitochondrial membrane damage, and cytochrome in mitochondria is released to the outside of cells, which activates CASP3 and induces apoptosis.^[19]^ CASP3 has attracted more and more attention as a target for the treatment of some cancers, and the study of CASP3 inhibitors is one of the effective methods for the development of anticancer drugs.

GO and KEGG enrichment analysis showed that MSMY treatment of ALL may be related to the regulation of apoptosis, positive regulation of gene expression and protein binding. KEGG enrichment analysis involved in cancer, phosphatidylinositol 3 kinase-protein kinase B, MAPK and IL-17 signaling pathways. PI3K-Akt plays a key role in the prevention and treatment of ALL. Abnormal activation of PI3K-Akt is one of the most common pathogeneses of ALL. Down-regulation of PI3K-AKT can promote autophagy and apoptosis of ALL cells.^[20]^ MAPK signaling pathway is a process that transduces extracellular stimuli into cells and their nuclei and causes cellular biological responses. Ras/Erk signaling pathway is the most widely studied MAPK signaling pathway. Ras activates MAPK cascade and participates in the regulation of cell proliferation and differentiation. MAPK signal transduction pathway plays an important role in the pathogenesis of leukemia by mediating apoptosis inhibition signals to cause malignant transformation of cells, excessive proliferation and apoptosis inhibition.^[21]^ IL-7 has the ability to inhibit spontaneous apoptosis and promote the proliferation of ALL cells in vitro, which may be achieved by activating PI3K/Akt signaling pathway, up-regulating Bcl-2 and down-regulating P27kip1.^[22]^ Therefore, the treatment of ALL with MSMY may be highly related to signaling pathways, and relevant molecular experiments should be strengthened to explore the mechanism of action.

## 5. Conclusion

In this study, on the premise that the clinical efficacy of MSMY in the treatment of ALL is confirmed, it lays the foundation for the following research, and uses the protein interaction network to explore the internal molecular mechanism of MSMY in the treatment of ALL. The results showed that the intrinsic mechanism of MSMY intervention in ALL may be related to the activation of PI3K-Akt, MAPK, IL-17 and other related signaling pathways mediated by MSMY core activity, which affects cell apoptosis and gene expression, so as to intervene in ALL.

The chemical composition of TCM compound is complex, showing the characteristics of multi-component, multi-target and multi-way. With the development of systems biology, network pharmacology provides a feasible research method for exploring the internal principle of TCM active ingredients intervening diseases and the construction of TCM multi-target precise treatment mode, which plays a promoting role in the development of TCM.

## Author contributions

**Conceptualization:** Dongyu Guo, Jing Hao.

**Data curation:** Dongyu Guo, Jing Hao.

**Formal analysis:** Dongyu Guo, Mengyu Gu.

**Investigation:** Yuting Zhao.

**Methodology:** Dongyu Guo, Mengyu Gu, Fuquan Du.

**Project administration:** Dongyu Guo, Fuquan Du.

**Resources:** Dongyu Guo, Yuting Zhao, Mingjie Gao.

**Software:** Fuquan Du, Yuting Zhao.

**Supervision:** Dongyu Guo, Mingjie Gao.

**Validation:** Dongyu Guo, Yuting Zhao, Mingjie Gao.

**Visualization:** Dongyu Guo, Mingjie Gao.

**Writing – original draft:** Dongyu Guo, Jing Hao.

**Writing – review & editing:** Dongyu Guo.
